# Enteroparasite and vivax malaria co-infection on the Brazil-French Guiana border: Epidemiological, haematological and immunological aspects

**DOI:** 10.1371/journal.pone.0189958

**Published:** 2018-01-02

**Authors:** Rubens Alex de Oliveira Menezes, Margarete do Socorro Mendonça Gomes, Anapaula Martins Mendes, Álvaro Augusto Ribeiro D’ Almeida Couto, Mathieu Nacher, Tamirys Simão Pimenta, Aline Collares Pinheiro de Sousa, Andrea Regina de Souza Baptista, Maria Izabel de Jesus, Martin Johannes Enk, Maristela Gomes Cunha, Ricardo Luiz Dantas Machado

**Affiliations:** 1 Postgraduate Program in the Biology of Infectious and Parasitic Agents, Federal University of Pará (UFPA), Belém, Pará State, Brazil; 2 Laboratory of morphofunctional and parasitic studies with impact on health (LEMPIS), Federal University of Amapá (UNIFAP), Macapa, Amapá State, Brazil; 3 Amapá State Secretary of Health (SESA)/Amapá Central Laboratory, Macapá, Amapá state, Brazil; 4 UNIFAP/Oiapoque Binational Campus, Federal University of Amapá, Oiapoque, Amapá State, Brazil; 5 Centre d’Investigation Clinique, CIC INSERM 1424, Centre Hospitalier de Cayenne, Cayenne, French Guiana; 6 Postgraduate Program in Neuroscience and Cell Biology, UFPA, Belém, Pará State, Brazil; 7 Evandro Chagas Institute/Brazilian Secretariat of Health Surveillance (SVS)/Brazilian Ministry of Health (MS), Ananindeua, Pará State, Brazil; 8 Fluminense Federal University, Niterói, Rio de Janeiro State, Brazil; 9 Laboratory of Microbiology and Immunology, Federal University of Pará (UFPA), Belém, Pará State, Brazil; Institut Pasteur, FRANCE

## Abstract

Malaria-enteroparasitic co-infections are known for their endemicity. Although they are prevalent, little is known about their epidemiology and effect on the immune response. This study evaluated the effect of enteroparasite co-infections with malaria caused by *Plasmodium vivax* in a border area between Brazil and French Guiana. The cross sectional study took place in Oiapoque, a municipality of Amapá, on the Amazon border. Malaria was diagnosed using thick blood smears, haemoglobin dosage by an automated method and coproparasitology by the Hoffman and Faust methods. The anti-PvMSP-1_19_ IgG antibodies in the plasma were evaluated using ELISA and Th1 (IFN-γ, TNF-α and IL-2), and Th2 (IL-4, IL-5 and IL-10) cytokine counts were performed by flow cytometry. The participants were grouped into those that were monoinfected with *vivax* malaria (M), *vivax* malaria-enteroparasite co-infected (CI), monoinfected with enteroparasite (E) and endemic controls (EC), who were negative for both diseases. 441 individuals were included and grouped according to their infection status: [M 6.9% (30/441)], [Cl 26.5% (117/441)], [E 32.4% (143/441)] and [EC 34.2% (151/441)]. Males prevailed among the (M) 77% (23/30) and (CI) 60% (70/117) groups. There was a difference in haemoglobin levels among the different groups under study for [EC-E], [EC-Cl], [E-M] and [Cl-M], with (p < 0.01). Anaemia was expressed as a percentage between individuals [CI-EC (p < 0.05)]. In terms of parasitaemia, there were differences for the groups [CI-M (p < 0.05)]. Anti-PvMSP-1_19_ antibodies were detected in 51.2% (226/441) of the population. The level of cytokines evaluation revealed a large variation in TNF-α and IL-10 concentrations in the co-infected group. In this study we did not observe any influence of coinfection on the acquisition of IgG antibodies against PvMSP119, as well as on the profile of the cytokines that characterize the Th1 and Th2 patterns. However, co-infection increased TNF-α and IL-10 levels.

## Introduction

In Brazil, over 99% of reported malaria cases occur in the Amazon region [1,2]. The cases recorded outside the Amazon region are imported from the Amazon states or from other countries. There are rare indigenous accounts that have been restricted to the Atlantic Forest in the south-eastern part of the country [3]. Socioeconomic and environmental conditions favour disease transmission and vector survival [4]. In recent years, *Plasmodium vivax* has been the most prevalent species in the country, accounting for approximately 80% of episodes, while *Plasmodium falciparum* is responsible for approximately 20%, and *Plasmodium malariae* is rarely detected. Historically, severe clinical *P*. *vivax* cases have been rarely reported[5]. However, recent studies emphasize the association of this species with clinical complications and fatal cases, which is a cause for public health concern [5–7].

Currently, it is estimated that more than a third of the world's tropical and sub-tropical population is infected with one or more enteroparasites [8]. Human infections with these organisms remain prevalent in countries where the malaria parasite is also endemic [9,10], and they are one of the most important public health issues in the world [11,12]. Malaria and intestinal parasite co-infections are widespread, and both have similar geographical distributions and overlap in developing countries [9,13].

Although *Plasmodium* and helminth co-infections are prevalent in tropical countries, the effect of their interactions remains unclear [14]. Some studies report that individuals who are infected with helminths are susceptible to *Plasmodium* infection [15,16], resulting in an increase in circulating gametocytes [17], a reduction in haemoglobin levels [18], the suppression of acute clinical manifestations [19] and an increased risk of malaria transmission [17,20,21]. It is known that increased incidence and prevalence of malaria can affect the development of *P*. *vivax* and *P*. *falciparum*-mixed infections and that parasite diversity may be greater in patients who are infected with helminths [22,23]. The evolutionary implications of co-infection may also extend to the reproduction of helminths, which are interested in protecting their hosts to survive and reproduce [24].

These associations between helminthiases and malaria are well documented in malaria-endemic regions of Africa and Asia, especially where malaria is caused by *P*. *falciparum*. However, the protective effect against malaria from *P*. *vivax* has also been observed in two studies outside Brazil [18,19]. In the Brazilian Amazon region, intestinal helminthiases were associated with protection against reduced haemoglobin levels during *P*. *vivax* malaria episodes in a population of children in the city of Manaus, Amazonas State [25]. In addition, in the municipality of Porto Velho in the State of Rondônia, co-infection with enteroparasites did not affect the immune response pattern to *vivax* malaria [26,27], but differences were observed between the haemoglobin levels of malaria patients and individuals who were not infected by enteroparasites [26].

The municipality of Oiapoque is in the French Guiana border region, and it has a close relationship with local social health determinants. It also has an intense population flow. The objective of this study was to evaluate the effect of enteroparasite co-infection on malaria caused by *Plasmodium vivax* in this region of the Brazilian Amazon.

## Methods

### Study area

A cross-sectional study was conducted in the municipality of Oiapoque, Amapá State, in northern Brazil, on the western border of the Amazon region. It has an Annual Parasite Index that designates it as at high risk of malaria transmission, and it is located in the northern part of the state of Amapá. Due to the border with the municipality of Saint Georges de l’Oyapock in French Guiana, there is an intense population flow related to trade, mining, tourism and social life, which also facilitates the spread of communicable diseases.

### Inclusion criteria

Participants were required to meet the following inclusion criteria: (1) signed consent form, (2) being native to the study area, (3) being over seven years of age, (4) consenting to blood collection and (5) providing a faecal sample.

After compliance with the inclusion criteria, in addition to the median, subjects were grouped according to age following the World Health Organization categories, as follows: “children” were those aged 0 to 9 years, “adolescents” those between 10 and 19 years of age, “adults” individuals aged 20 to 59 years and “seniors” those over 60 years of age.

### Sample

A total of 441 participants formed the study population, with the sample collection taking place over one year between the months of November 2014 and November 2015. Data collection was performed by passive detection in the municipality's Basic Health Units, following phenotypic diagnoses using thick blood smears and prior to treatment. For the uninfected group, samples were also collected in the same municipality so that the participating individuals were froma similarbackground exposure conditions. After a parasitological evaluation, the subjects were divided into the following four groups: 1) patients with an intestinal parasite and malaria co-infection (n = 117); 2) patients with malaria who were negative for intestinal parasites (n = 30); 3) patients without malaria who were positive for enteroparasites (n = 143); and 4) patients who were negative for both (n = 151). Epidemiological data such as the age, gender and number of previous episodes of malaria were obtained during interviews and from medical records.

### Ethical considerations

The present work is an integral part of the project "Coinfection of intestinal helminthiasis and susceptibility to infection by *Plasmodium vivax* and *Plasmodium falciparum* on the Franco-Brazilian border", which was certified by the Research Ethics Committee of the Federal University of Amapá - CEP/UNIFAP, on December 20, 2013, protocol n° 18740413.7.0000.0003, as being in accordance with the Ethical Principles in Human Experimentation, adopted by the National Committee of Ethics in Research—CONEP. The research subjects were invited to participate by Free and Informed Consent signature while the inclusion of those under 18 years of age was conditioned to their parents or guardians signature of a Free and Clarified Consent. Fingerprinting was used to group non-literates. All patients identified as positive for malaria received chemotherapy treatment asprescribed by the medical staff of the present study, provided by the Brazilian Ministry of Health, while those diagnosed with intestinal parasitoses were referred to medical treatment in one of the Oiapoque mucicipality Health Units.

### Collection and laboratory analysis

#### Blood collection

Blood samples were collected by venipuncture. Ten millilitres of venous blood was collected from each patient. Four millilitres was dispensed into a tube containing EDTA (ethylenediamine tetraacetic acid) (Beckton & Dickson, USA) to perform haematological analysis and to prepare thick blood smear slides, and 6 mL was dispensed into a tube (Becton & Dickson, USA) with no anticoagulant for immunological analysis. After this collection, each slide was maintained at room temperature to dry the blood drop. The slides were subsequently stained and analyzed using light microscopy (Nikon, Japan) according to the protocol described by the World Health Organization.

#### Malaria diagnosis

The slides were prepared by following the Walker technique (methylene blue and Giemsa) [28] and evaluated by a local microscopist from the municipality’s malaria diagnostic station. Direct parasite counting was performed by estimating the parasitaemia using a semi-quantitative evaluation, which recorded the parasitaemia interval per μL of blood from 200 fields. The number of counted parasites was multiplied by 5 [29]. These results were subsequently confirmed in the Amapá Border Laboratory (LAFRON/AP) and by the endemic quality control section of the Amapa Central Laboratory (LACEN/AP).

For the sexual form count (gametocytaemia), the thick blood slides were reviewed for gametocyte quantification in 100 leukocytes. The conversion to gametocytes/μL was performed by calculation using a reference value of 8000 leukocytes/mm^3^, and the gametocytes were counted directly on the slide (N° gametocytes X N° of leukocytes (8000)/100 = gametocytes per μL). This procedure is similar to the diagnostic methods recommended by the Clinical and Laboratory Standards Institute (CLSI) guidelines of the United States Centers for Disease Control (CDC) and the World Health Organization (WHO) guidelines. The count was subsequently confirmed in LAFRON/AP and sent to LACEN/AP's endemic quality control section.

#### Plasmodia molecular diagnosis

In order to confirm parasitological diagnosis, DNA was extracted from whole blood using the Easy-DNATM kit (Invitrogen, Carlsbad, California, USA) and the QIAamp® DNA Blood Kit (Qiagen, Inc., Chatsworth, CA) followed by nested PCR, as previously described by Snounou et al. (1993) [30].

#### Haemoglobin dosage

Anemia was evaluated according to the diagnostic standards recommended by the World Health Organization through hematocrit (hemocytes and hemoglobin) indices with slight variations according to age and sex. The haemoglobin concentration was measured in venous blood using the Oiapoque Hospital's automated equipment (Mindray-BC-3000plus). Anemia was defined with haemoglobin reference values. The haematological parameters evaluated were the total number of erythrocytes (RBC; reference range: male 4.5–6.5 x 106/μL, female 3.9–5.6 x 106/μL and children aged 7–11 years 4.5–4.7 x 106/μL) and haemoglobin levels (Hb; males ≥ 13 g/dL, females ≥ 12 g/dLand children ≥ 11 g/dL). Individuals were considered anemic when their haemoglobin blood levels were ≤ 13 g/dL for males, ≤ 12 g/dL for females and ≥ 11 g/dL for the children.

#### Evaluation of anti-PvMSP-1_19_ IgG antibodies

The evaluation of total IgG antibodies against MSP-119 was performed at the Federal University of Pará, following the protocol of Cunha et al. (2001) [31]. The recombinant protein (His6-MSP-119) from *P*. *vivax* MSP-1 (Belém strain) was expressed in *Escherichia coli*. The 96 wells Enzyme Linked Immuno Sorbent Assay (ELISA) plates (Costar, Corning Inc., NY, USA) were sensitized with 50 μL of His-MSP-119 protein (2 μg/mL) diluted in 0.05M carbonate buffer pH 9.0, for 16 hours at room temperature (RT). Subsequently, the plates were washed with PBS/Tween 0.05% and blocked with 200 μl of 5% PBS skin milk. After 2 hours at 37°C, the plates were washed and 50 μl of the patient’s plasma, diluted 1:100 in PBS/milk, were added in each well, in duplicate, and the plates were incubated for 16 hours (RT). After rinsing the plates with PBS 0.05% Tween, 50 μl of peroxidase-linked human IgG (DAKO polyclonal rabbit, Denmark) conjugate diluted 1:10,000 in 5% milk PBS was added to each well, and the plates were incubated for 2 hours at room temperature. After a final wash with PBS 0.05% Tween, 100 μl of OPD (ortho-phenylenediamine) (1 mg/ml) diluted in phosphate-citrate buffer (NaH2PO4 0,2 M, C6H8O7 0,2 M, pH 5,0) containing 0.03% hydrogen peroxide were added to the plates. This reaction was kept in the dark and quenched after 10 minutes by the addition of 25 μl of H2SO4 4 N in each well. Optical density (OD) was quantified in ELISA reader (EL800 Bio Tek, Winooski, USA) at a wavelength of 490 nm. The cutoff point was established by the mean OD of 56 plasma samples from individuals with no history of malaria, from the Center for Hemotherapy and Hematology of Pará (HEMOPA) residing in Belém-Pará, plus three standard deviations. The reactivity index (IR) was determined by dividing the OD value of the sample by the cutoff point (OD/OD cutoff value). IR values were used to estimate the concentration of IgG antibodies.

#### Cytokine dosage

A CBA (cytometric bead array) Kit (BD) was used in this study to quantify cytokines IL-2, INFγ and TNF (Th1) and IL-4, IL-5, and IL-10 (Th2) in the same sample. Six bead populations with distinct fluorescence intensities were conjugated with a capture antibody specific for each cytokine. They were mixed to form the CBA and read on a FACSCanto II-type flow cytometer (Becton Dickinson, San Jose, CA) that was previously calibrated with "setup beads", incubated with fluorescein isothiocyanate (FITC) or phycoerythrin (PE) according to the manufacturer's recommendations. A standard curve was performed for each cytokine and analyzed using FACSDiva software (Becton Dickinson, San Jose, CA, USA). The bead populations were displayed according to their respective fluorescence intensities, from dimmer to brighter. In the CBA, the cytokine capture beads were mixed with detection antibody conjugated with PE fluorochrome and then incubated with the test samples to form a "sandwich" test. The acquisition tubes were prepared with 50 μL of sample, 50 μL of bead mix and 50 μL of Th1/Th2 PE detection reagent (Human Th1/Th2 PE Detection Reagent/1 vial, 4 mL). The same procedure was performed to obtain the standard curve. The tubes were homogenized and incubated for three hours at room temperature in the dark. The results were presented on graphs and in tables using FCAP Array 3 software (Becton Dickinson, San Jose, CA, USA). Raw MFI (media fluorescence intensity) values were quantified for each cytokine. The values were expressed in pg/mL for each cytokine in comparison to the standard curve. Three hundred events were considered for each cytokine.

#### Fecal diagnosis

All individuals were provided with two sterile plastic containers and asked to provide faecal samples to be collect in the morning as follows: one without any solution and another with a preservative solution of 10% formaldehyde. For the negative cases three fecal samples were requested on alternate days to increase the detection sensitivity and to also to double check parasite nullity by negative slides. Fecal samples were prepared using the technique and/or methods of Hoffman-Pons-Janer and Faust. For each sample, two slides were examined for detection of parasites by two investigators with identification experience, using optical microscopy (Nikon, Japan) with magnifications of 100X and 400X. All fecal analyzes were performed in a private laboratory in the municipality of Oiapoque/AP.

### Statistical analysis

Each volunteer's epidemiological data results, which were obtained from the questionnaire, was stored in an Epi-Info 3.5.1 database (CDC, Atlanta, GA, USA). The values for each group (malaria, co-infected, enteroparasite and endemic control), as well as the subgroups (helminths, protozoa and helminth-protozoa association), were expressed as a percentage. The age, residence time in Oiapoque (years) (RT), number of previous malaria episodes (NPE), period since last malaria (months) (PLM), haemoglobin levels (g/dL), parasitaemia (parasites/L) and gametocytes were expressed as medians (1st and 3rd Quartile) using BioEstat 5.3 statistical software. The differences between groups with regard to age, RT, NPE, PLM and haemoglobin were calculated using Tukey's test based on a one-way analysis of variance (ANOVA). The Kruskal-Wallis-Dunntest was used to calculate the degree of anaemia.

The statistical tests used here were chosen by considering the size and type of each variable and number of evaluated groups. The differences in the parasitaemia and the gametocytes between the malaria and co-infected groups were calculated using the *Wilcoxon-Mann-Whitney test*. For the PvMSP1_19_ reactivity index (RI) among the studied groups, multiple correlations were performed using the non-parametric Kruskal-Wallis test, followed by Dunn's post-test. The data were expressed in box plot format (minimum to maximum values, P25%-P75% and median). Significant differences were estimated using the median values for each group; those with p ≤ 0.05 were considered significant.

For the cytokine expression analysis, the significance level was obtained by comparing the cytokine concentrations of the studied groups using the GraphPad Prism program, version 6.0 (GraphPad Software, San Diego, CA, USA). An analysis to uncover the correlation between the groups was performed using the non-parametric Kruskal-Wallis test followed by Dunn's post-test. The data were expressed in box plot format (minimum to maximum values, P25%-P75% and median). Significant differences were estimated using the median values for each group, and those with p ≤ 0.05 were considered significant.

## Results

### Prevalence of malaria and enteroparasites

Table 1 summarizes the individual results according to their infection status. In summary, 6.9% (30/441) of the individuals were infected with malaria alone (M); 26.5% (117/441) of the individuals were co-infected with malaria and enteroparasites (CI); 32.4% (143/441) of the individuals were infected with enteroparasites only (E); and 34.2% (151/441) of the individuals had a negative diagnosis for malaria and enteroparasites/endemic control (EC). The (CI) and (E) groups consisted of subjects who were positive for intestinal parasitic infection, with helminths only (H), protozoa only (P) or an association of helminths and protozoa (P+H) (S1 Table).

**Table 1:** Distribution and number of individuals among groups and subgroups according to the malaria and intestinal parasite diagnosis.

Groups	Subgroups	Description	n	%
Malaria (M)		Individuals infected with *Plasmodium vivax* only	30	6.9
Co-infected (CI)	Helminths (H)	Individuals co-infected with *Plasmodium* and helminths only (H)	54	12.2
	Protozoa (P)	Individuals co-infected with *Plasmodium* and protozoa (P) only	39	8.9
	Helminths + Protozoa (P+H)	Individuals co-infected with *Plasmodium* and helminths + protozoa (P+H)	24	5.4
Total (CI)			117	26.5
Enteroparasite (E)	Helminths (H)	Individuals infected with helminths only (H)	63	14.2
	Protozoa (P)	Individuals infected with protozoa only (P)	68	15.4
	Helminths + Protozoa (P+H)	Individuals infected with helminths and protozoa (P+H) only	12	2.8
Total (E)			143	32.4
Endemic Control (EC)		Individuals negative for malaria and intestinal parasite diagnosis	151	34.2
Total			441	100

Groups: malaria (M), co-infected (CI), enteroparasite (E) and endemic control (EC).

Subgroups CI and E: helminths (H), protozoa (P) and association of helminths and protozoa (P+H).

### Characteristics of the studied groups

Table 2 summarizes the characteristics of the studied groups. Males represented the majority of individuals who were infected with malaria in both the M group, 77% (23/30), and CI group, 60% (70/117). When using a one-way ANOVA with Tukey's test, it could be observed that the mean age of the CI (p < 0.01) and E (p < 0.05) groups differed from that of the (EC) group. However, this trend did not occur between the other groups (S1 and S2 Tables).

**Table 2:** Epidemiological and haematological data for the studied groups.

	Malaria-Positive N = 147		Malaria-Negative N = 294	
	Malaria (M)^a^ N = 30	Co-infected (CI)^b^ N = 117	Enteroparasites (E)^c^ N = 143	Endemic control (EC)^d^ N = 151
Category n (%)				
Male	23 (77)	70 (60)	56 (39)	88 (58)
Female	7 (23)	47 (40)	87 (61)	63 (42)
Age	29 (12–55)	29 (7–66)^d^*	25 (8–74)^d^*	19 (10–60)
RT	29 (12–79)^d*^	26 (7–66)^d^*	25 (8–65)^d^*	19 (10–60)
NPE	4 (2–10)	5 (0–17)	4 (0–17)	4 (1–16)
PLM	9 (5–14)	8 (0–15)^c^*^d^*	9 (0–17)	9 (4–18)
Haemoglobin (g/dL)	13.8 (11.7–17)^b^*^c^*	13.2 (7.7–18.2)^d^*	12.4 (9.4–16.7)^d^*	13.7 (9.4–16.7)
Anaemia (%)	10% (3/30)	30.8% (36/117)^d^*	43.3% (62/143)	10.6% (16/151)
Parasitaemia (par./*μ*L)	2750 (60–16.000)^b^*	1000 (25–30.000)	(—)	(—)
Gametocytes	250 (0–6000)	70 (0–6000)	(—)	(—)

n (%): number of samples (percentage) in each category

Values expressed as medians (25–75%): age, residence time (years) in Oiapoque (RT), number of previous malaria episodes (NPE), period (months) since last malaria (PLM), haemoglobin levels (g/dL), parasitaemia (parasites/L) and gametocytes.

The differences between the groups with regard to their age, RT, NPE, PLM and haemoglobin were calculated using Tukey's test and based on a one-way ANOVA.

Individuals with haemoglobin levels ≤ 13 g/dL for men, ≤ 12 g/dL for women and children ≥ 11 g/dLwere considered to have anaemia, according to the Kruskal-Wallis-Dunn test.

Differences in parasitaemia and gametocytes between the malaria and co-infected groups were calculated using the Wilcoxon Mann-Whitney test.

^a^Difference between indicated group and the malaria group.

^b^Difference between the indicated group and the co-infected group.

^c^Differences between the indicated group and the enteroparasite group.

^d^Differences between the indicated group and the endemic control.

Statistical differences in epidemiological parameters were expressed as * p < 0.05.

We also observed differences in the mean residence time (RT) for groups E, CI and M (p < 0.05) when compared to the EC group. Regarding the number of previous malaria episodes (NPE), there were nodifferences between groups (p = 0.0716). When analyzing the period since last malaria (PLM) variable, we observed differences between the CI and EC groups (p < 0.01) and between the E and CI groups (p < 0.01).

Analyses of some haematological data showed that the mean haemoglobin levels differed between the groups EC and E (p < 0.01), EC and CI (p < 0.01), E and M (p < 0.01) and M and CI (p < 0.01). However, there was no significant difference when comparing groups EC and M and E and CI. Anaemia was expressed as a percentage, and the Kruskal-Wallis-Dunntest found a difference between the CI and EC individuals (p-value = 0.0224). With regard to parasitaemia (count of *Plasmodium vivax*parasites), there were differences between the CI and M groups (*p* = 0.0152), as calculated by the Wilcoxon-Mann-Whitney test. However, when using the same test to quantify the *Plasmodium vivax* gametocytes, no statistically significant differences were observed between the CI and M groups (*p* = 0.0819) (S1 and S2 Tables).

### Anti-PvMSP1_19_ IgG responses to *Plasmodium vivax*

**Fig 1:**
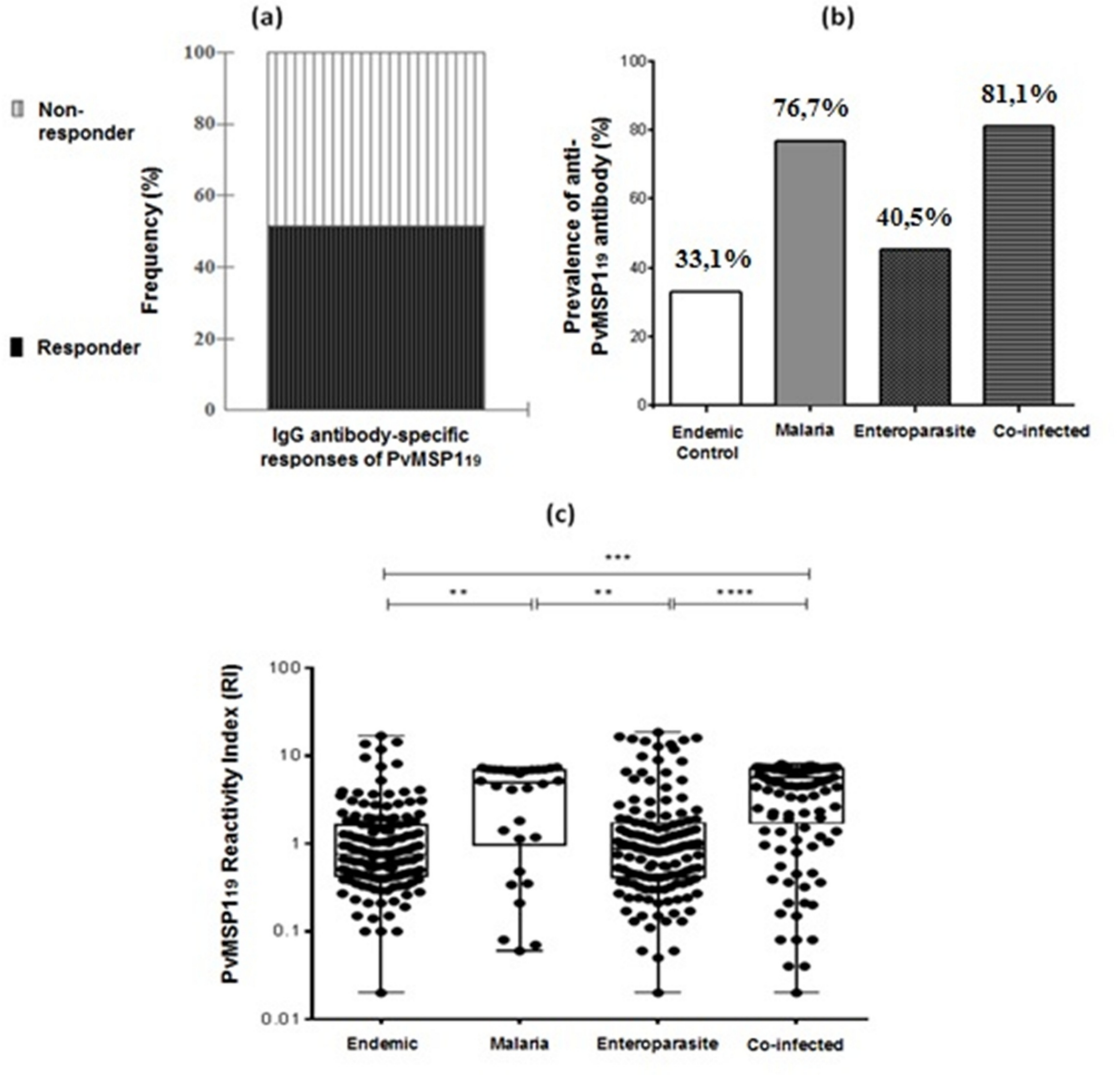
(a) Frequency-specific antibody response to PvMSP-1_19_, as determined by ELISA. The subjects were grouped into responders and non-responders to the recombinant protein. (b) Prevalence of anti-PvMSP-_19_ IgG antibodies in the studied groups. (c) PvMSP-1_19_ reactivity index (RI) between the studied groups as expressed in box plot format, with individual data shown as points. Multiple correlations were made using the nonparametric Kruskal-Wallis test followed by Dunn’s post hoc test (minimum to maximum values, P25%–P75% and median); significant differences were estimated using the median values for each group, and those with p < 0.05 were considered significant. ** p < 0.05, *** p = 0.001 and **** p < 0.001.

The percentage of individuals in the investigated population that possessed naturally acquired anti-PvMSP-1_19_ IgG-1 antibodies is shown in (Fig 1). Responders represented 51.2% (226/441) of individuals (Fig 1A). The presence of anti-PvMSP-1_19_ IgG antibodies among the groups showed a higher prevalence of 81.1% (95/117) in the CI group. The lowest prevalence was in the EC group, with 33.1% (50/151) (Fig 1B). To support the data (Fig 1C), each individual's data were shown as a point and expressed in box plot format. Significant differences were observed, as estimated by the median values for each group (a nonparametric Kruskal-Wallis test followed by Dunn’s post-test). These values were considered significant, where p < 0.05(S1 Fig).

**Fig 2:**
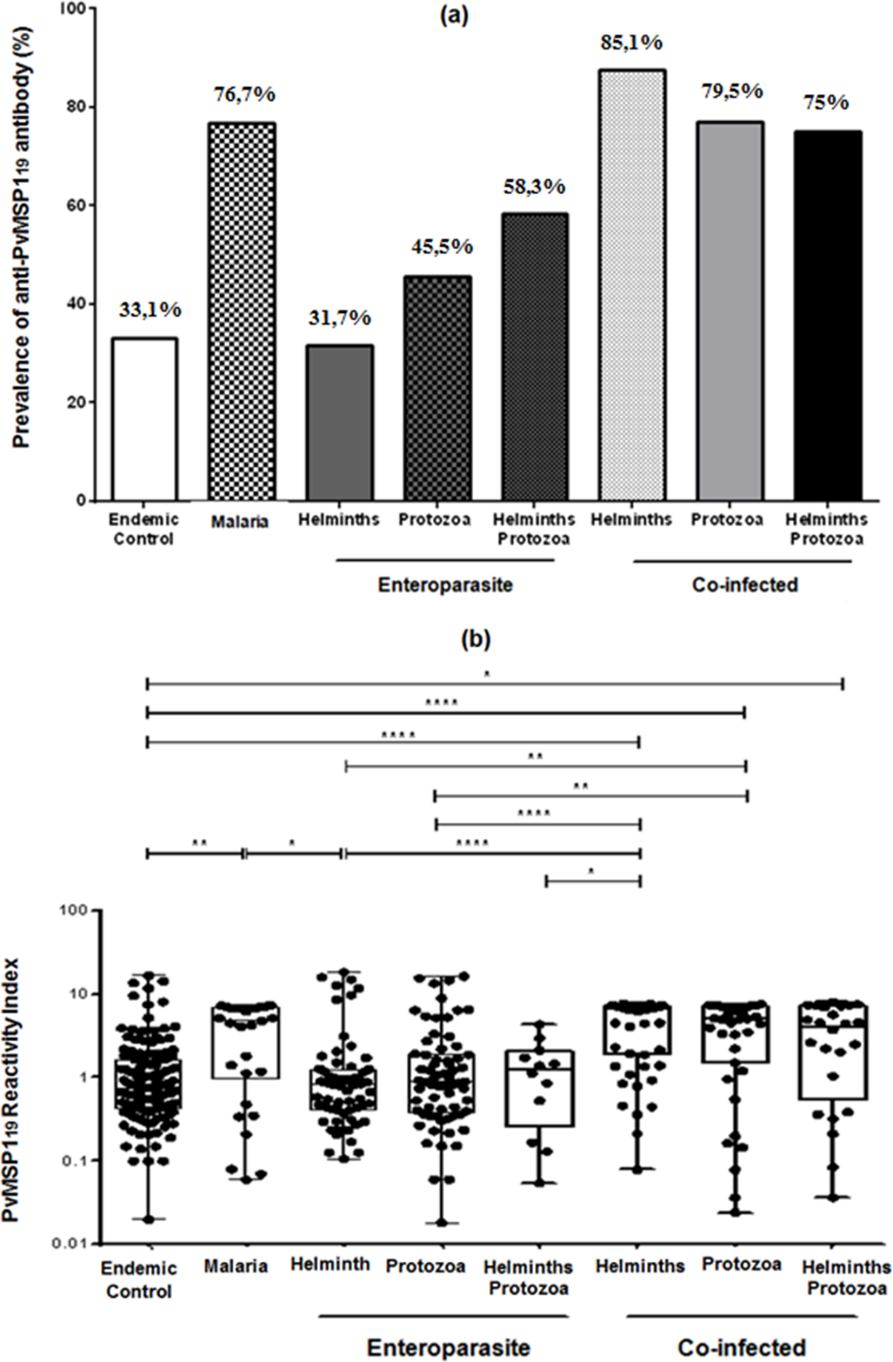
(a) Prevalence of recombinant PvMSP-1_19_ antigens in the studied groups, according to the intestinal parasitic infection. The (enteroparasite) and (co-infected) groups have subgroups of individuals that were infected with helminths, protozoa and protozoa and helminths together. (b) The PvMSP1_19_ reactivity index (RI) between the endemic control, malaria, enteroparasite (helminths, protozoa and protozoa+helminths) and co-infected (helminths, protozoa and protozoa+helminths) groups. Multiple correlations were made using the nonparametric Kruskal-Wallis test followed by Dunn's post hoc test. Data are expressed in box plot format (minimum to maximum values, P25%–P75% and median), with all individual data shown as points. Significant differences were estimated using the median values for each group, and those with p < 0.05 are considered significant. * p = 0.05, ** p < 0.05, *** p = 0.001 and **** p < 0.001.

Fig 2 shows the seroprevalence in terms of the frequency of anti-MSP1_19_-specific IgG antibodies as distributed across the four study groups as well as the reactivity index, which specified and subdivided the CI and E groups. (Fig 2A) shows that the helminths were most prevalent in the CI group, with 85.1% (46/54). The highest values in group E were found in the protozoa and helminth association subgroup, with 58.3% (7/12). (Fig 2B) expresses the PvMSP-1_19_ reactivity index (RI) among the studied groups structured as in (Fig 2A). The data are expressed in box plot format (minimum to maximum values, P25%-P75% and median), with each individual's data shown as a point. Significant differences were observed in each group’s median values, and those with p < 0.05 were considered significant. ** p < 0.05, *** p = 0.001 and **** p < 0.001 (S1 Fig).

### Circulating cytokine profile in the population's plasma

**Fig 3:**
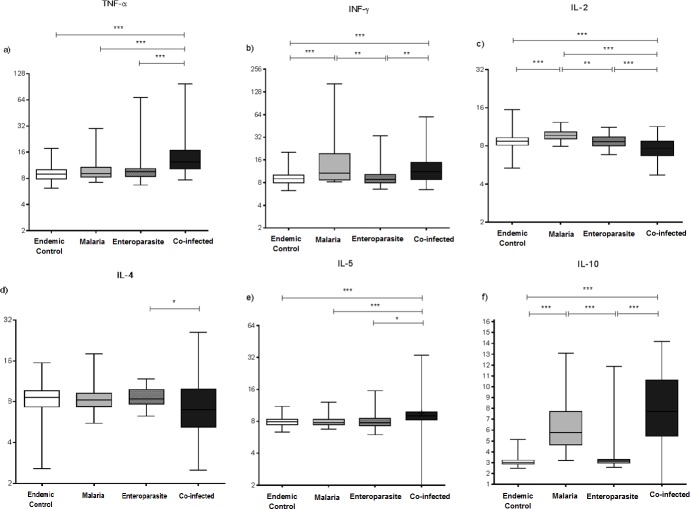
Serum levels of TNF-α (a), IFN-γ (b), IL-2 (c), IL-4 (d), IL-5 (e) and IL-10 (f) cytokines in pg/mL among the following groups: endemic control, malaria, enteroparasite and co-infected groups. Multiple correlations were made using the non-parametric Kruskal-Wallis test followed by the Dunn's post hoc test. Data are expressed in box plot format (minimum to maximum values, P25%–P75% and median). Significant differences were estimated using the median values for each group, with p < 0.05 being considered significant. * p = 0.05, ** p < 0.05 and *** p = 0.001.

A total of 130 of the 441 individuals analysed at the start of the study were evaluated to verify the circulating cytokine profile in the population's plasma, from groups M (n = 30), CI (n = 50), E (n = 50) and EC (n = 100). The TNF-α concentration was higher in the CI group than in the other groups. The median values differed for the CI-E, M-CI and CI-EC groups (p = 0.001) (Fig 3A) (S2 Fig).

Additionally, the IFN-γ levels were higher in group M than in the other groups. Comparisons of the M-EC (p = 0.001), M-E (p < 0.05), E-CI (p < 0.05) and EC-CI (p = 0.001) groups were statistically significant (Fig 3B). Increased IL-2 concentration values were observed in the EC group. Statistically significant differences were observed for the EC-CI (p = 0.001), EC-M (p = 0.001), M-CI (p = 0.001), M-E (p < 0.05) and CI-E (p = 0.001) groups (Fig 3C) (S2 Fig).

The IL-4 concentration was higher in the CI group, and there was a statistically significant difference for CI-E (p = 0.05). Comparisons between the remaining groups were not statistically significant (Fig 3D). IL-5 showed high concentrations in the CI group, and comparisons of the CI-E (p = 0.05), CI-M (p = 0.001) and EC-CI (p = 0.001) groups were significant (Fig 3E). With respect to the IL-10 cytokine profile, we can observe increased concentrations for groups CI and M. There were statistically significant differences for groups CI-E, CI-EC, M-E and M-EC (p = 0.001) (Fig 3F) (S2 Fig).

## Discussion

Microorganism co-infections are largely unexplored, and their effects can be either favourable or harmful to the human host. Some studies have explored the effects of helminth co-infections on the dynamics of *P*. *falciparum* malaria transmission and its correlation with anaemia [26,27]. In Brazil, recent studies have described the haematological and immunological profiles of *vivax* malaria and intestinal parasite co-infections [25–27] in two areas of Brazil's western Amazon. We are the first to study the effect of intestinal parasites on biological aspects of malaria in individuals who were naturally infected with *P*. *vivax* in a malaria-endemic area located on the Brazilian border with French Guiana.

The parasitological findings of this study show a higher prevalence of individuals who were infected only with E (32.4%), followed by the CI group (26.5%). Group M had the lowest prevalence (6.9%). The CI and M groups were predominant in the male population, emphasizing their geographical peculiarities and aspects related to the primary constraints and health determinants in the border region related to work activities, more specifically, the illegal mining (gold mining) and agricultural activities prevalent in the region where contact with the vector and soil is frequent.

Of all individuals included in this study, the minority was composed by children (2.2%;10/441) and seniors (1.4%; 6/441), while 37% (163/441) and 59.4% (262/441) were adolescents and adults, respectively. Likewise, the wide age range of our study population probably did not affect the observed results, since children and seniors, expected to suffer greater variations in relation to the immunological response, only represented 3.6% of the total number of subjects. The present study’s research group was mainly composed of adults and young adolescents, belonging to a socioeconomically disfavored population stratum, mainly working in in activities related to mining.

The results show that individual haemoglobin levels differ between the CI and M groups. Anaemia was more prevalent in the CI and E individuals, with significant differences in the plasmodial parasite load between the CI and M groups. This haematological change observed between the groups emphasizes that anaemia is multifactorial and is a frequent early manifestation of malaria that contributes to disease severity, especially in the context of concurrent infections [32–34]. This disease leads to the destruction or sequestration of erythrocytes, abnormal erythropoiesis and blood loss arising from eventual coagulopathy [35–37]. Additionally, cytokine polymorphisms have been associated with susceptibility to severe malarial anaemia, disturbing erythropoiesis [38,39]. Studies have shown that a number of co-infections increase susceptibility to anaemia because they exacerbate the inflammation caused by malaria [40,41]. Helminth infections are associated with the increased likelihood of development and the greater severity of anaemia in *Plasmodium* infection [40] as well as genetic changes—haemoglobinopathies—and nutritional deficiencies [42].

The molecular mechanisms underlying malarial anaemia are largely unknown, although it is accepted that the disease is complex and multifactorial and has characteristics in common with acute malaria [42,43]. In this context, the results of this study indicate that the effect of enteroparasitic infection on the prevalence and severity of anaemia caused by *vivax* malaria still requires further investigation. These differences may be explained by the fact that in a co-infection, there may be a negative interaction between parasites, characterized by a protective effect on the individual and a suppressive effect on one of the pathogens or a positive interaction in which an infection is facilitated by the presence of the other parasite [43–45]. Thus, it is possible that *P*. *vivax* and intestinal parasite co-infection could modify the immune response profile against certain specific *Plasmodium* antigens.

Seroepidemiological studies to investigate the type and magnitude of the *vivax* malaria immune response in naturally exposed populations, in both Brazil [46–48] and around the world [49–52], have provided important information about the potential candidacy of immunogenic *P*. *vivax* antigens for use in creating an anti-malarial vaccine. The differences between antibody profiles reported in the present study indicate the importance of performing immunoepidemiological studies in different malaria-endemic areas, where transmission intensities and human genetic backgrounds are very distinct [53].

Studies on antibody responses to *Plasmodium* antigens offer a key process in the discovery and development of anti-malarial immunotherapy. Several studies have reported high antibody responses to P. *vivax* antigens in individuals who have been exposed to malaria infections [27,54]. In this study, anti-PvMSP-1_19_ IgG antibodies were detected in 51.2% (226/441) of the studied population, indicating the presence of an immunogenic protein, with results similar to those of other studies in the Amazon region [27,52–56]. Higher prevalence and specific antibody RIs were observed in the CI and M groups, reaching 81.1% (95/117) and 76.7% (27/30), respectively. As demonstrated in other endemic areas in the Brazilian Amazon, PvMSP-1_19_ appears as an immunogenic molecule during naturally acquired malaria infections [57–60].

Corroborating the findings in the Western Brazilian Amazon, the presence of enteroparasites also did not affect antibody responses to this antigen [27]. The reduced prevalence of IgG and RI in the group of individuals who were infected with intestinal parasites alone appears to be a general characteristic of this association, as in Rondônia, the prevalence of protozoa in *vivax* malaria-co-infected individuals was higher than that detected in the Oiapoque municipality, where geohelminths were more prevalent.

These results support those of other studies [61,62] that have suggested, theoretically, the possibility that malaria-helminth co-infection potentiates parasitic genetic diversity in individuals who are exposed to both infections [21,61,62]. Understanding this transmission dynamic and the evolutionary implications of populational *P*. *vivax* co-infection is very important for predicting the emergence and distribution of different strains of the parasite, which may differ in their virulence and drug resistance [63]. This finding is a major concern in these neglected border areas, where the usual practice of inadequate self-treatment contributes to an increased risk of developing resistance to antimalarial drugs.

In these regions, individuals from socio-economically disadvantaged populations frequently migrate between the two countries, especially those involved in illegal mining. This populational flow contributes to the practice of malaria self-medication and may influence plasmodial resistance to some antimalarial drugs. However, we did not find references in relation to the individual's immune response to the parasite, which, therefore, does not suggest important biases. It is worthy of note that in the Oiapoque municipality there are no de-worming policies, which could interfere in our study analysis.

Due to Oiapoque's local genetic diversity and the combined effects of the geographical structure of the border between Brazil and French Guiana in a regional, national and international context, there is a dispersion of *Plasmodium vivax* populations between the two countries [64]. Establishing *P*. *vivax*'s complex geographical pattern is important, both for evaluating diversity when encoding candidate antigens for vaccines and when formulating and structuring surveillance measures to control malaria.

In fact, helminths were the most prevalent enteroparasites in the CI (85.1%, 46/54) and E (58.3%, 7/12) groups. The reduction in the antibody prevalence and reactivity index in group E may be explained by the association (protozoa-helminths) and/or presence of intestinal protozoa. However, in the CI group, the IgG prevalence and reactivity index were not reduced and were similar to those in infected group M. These data confirm the non-importance of protozoa in the immune process during co-infection with *P*. *vivax*, as previously shown in another Brazilian Amazon region [27]. However, the diversity of enteroparasites meant that groups could not be formed with sample sizes large enough for researchers to be able to infer some sort of effect on co-infection.

The general trend in co-infection studies (helminths, malaria) is convergent and points to the possibility of increasing incidence and prevalence of malaria [65,66] and a tendency to reduce malaria symptoms [24,67]. This finding suggests that these patients might be less likely to seek treatment, making them a potential source of transmission [21]. In poor tropical areas, health determinants and constraints affect the population's health care and services sought when the disease is at an advanced stage.

In malaria, protective immunity is gradually acquired through the natural exposure of people living in areas where the disease is endemic, but this immunity is rapidly lost when exposure ceases, indicating that malaria's immunological memory is short term and requires constant exposure to the parasite [68,69]. Although infection by many enteroparasites was observed, the research revealed a prevalence of helminths, and it is generally believed that helminth infection may alter the host's natural immune response to *Plasmodium* due to the anti-inflammatory effect of helminth-induced cytokines [26].

Understanding the Th1/Th2 response pattern is of great importance for understanding the host's defense, and these antagonistic responses allow for the homeostasis of the immune system, which is important for parasite containment [70]. In cases in which an inflammatory pattern is prevalent, the disease tends to be more severe [5]. In this regard, the ability to measure numerous molecules and visualise inflammatory changes in individuals who are co-infected with malaria and enteroparasites is extremely important for advancing the understanding of the immune response to pathogens [26,27]. In this context, despite the importance of the PvMSP-1_19_ immunogenic molecule being present in response to malaria, other mediators, such as pro- and anti-inflammatory cytokines, also act in the elimination of plasmodial infections.

The individual analysis of mean serum cytokine levels in the studied population showed a broad variation in the serum concentrations of all the Th1 inflammatory profile cytokines (IFN-γ, TNF-α and IL-2) in the studied groups. Initially, we found that the expression of these cytokines exhibited a very similar profile between groups, with high levels for M and CI individuals. This observation suggests that only malarial infection, and not intestinal parasites, affected the increase in these levels. Elevated serum cytokine levels during infection have been reported previously, both in uncomplicated and severe malaria [71,72]. However, their participation in reducing the parasitic load remains controversial.

Some studies have shown the importance of the cytokine pattern and its interactions in the *vivax* malaria immune response [70]. Increased parasitaemia and immune responses during the course of the disease are factors that determine its severity [73]. Inflammatory patterns are rare, and few studies have evaluated the various cytokines present using the same method and the same group of malaria-infected patients [74]. This observation can also be corroborated when we observe the Th2 profile of anti-inflammatory cytokines (IL-4, IL-5 and IL-10), with increased serum concentrations in co-infected individuals. In fact, the high levels of these cytokines and their increased frequency among the groups infected with malaria (Cl and M) are consistent with the hypothesis that these cytokines play an anti-inflammatory role by inhibiting pro-inflammatory mechanisms after the initial stages of infection [75].

In the literature, IL-4 and IL-5 concentrations have been shown to confer protection against extracellular pathogens, including helminths [76]. Some studies have shown conflicting results, in which the concentrations of these cytokines depend on the host's parasitaemia levels [77]. Additionally, several studies focusing on IL-10 have shown that this cytokine plays an important role in the control of the antiparasitic response and tissue damage caused by this response, demonstrating its importance in regulating the immune response [78]. Furthermore, studies have shown that interactions between IL-6 and IL-10 cytokines are correlated with the parasitic load [79]. This study presents several variations in the Th1 cytokine expression (IFN-γ, TNF-α and IL-2), with similar medians among the studied groups, but with high and varying levels of each cytokine. The Th2 cytokine (IL-4, IL-5 and IL-10) medians differed from each other in the studied groups, showing high levels for individuals in the co-infected group.

Variations in the cytokine production and high Th1 (TNF-α) and Th2 (IL-4, IL-5 and IL-10) profile levels in the co-infected group therefore suggest that cytokines exhibit marked increases in their plasma concentrations when individuals are infected with *P*. *vivax*, with a shifting pattern inchanges associated with malaria and intestinal parasite co-infection. They seem to act in inflammatory processes in a nonspecific way, not exerting any effect on co-infection. However, only the IL-10 cytokine levels were affected in cases of malaria and intestinal parasite co-infection. Further wide-ranging studies are needed to evaluate the intestinal parasite species present in malaria-endemic areas and their relationship to the immune response of infected individuals.

These results may reflect the fact that the number of parasites in the bloodstream at a particular time may not always show the maximum immunogenic potential capable of inducing a host's immune response because some parasite antigens may be in immune system tissue and not just in the blood [80]. The overall magnitude of production of pro- and anti-inflammatory cytokines and the imbalance between them during co-infection have been proposed as important determinants in controlling or exacerbating malaria [81]. However, new immunological and molecular epidemiological studies are required to better understand the protective immune response scenario and outcome in *Plasmodium vivax* malaria. These studies will be essential in the development of vaccines and new immunotherapeutic targets.

The present study has some limitations, we acknowledge. First the cross sectional design is prone to bias and confounding and does not allow to infer causality. Secondly, the small sample size, notably in the malaria alone group, may not have been sufficient to detect some differences with other groups. Morevover, this evaluationdid not allow to look at the respective effects of different nematode species which may have contrasting effects. Likewise, the current results highlight the possibility that the enteroparasite and vivax malaria co-infection on the Brazil-French Guiana bordermark biological differences, until unexplained, towards the differential immune response and haematological profile of the host-parasite interaction in vivax malaria in Oiapoque, Amapá state. Aditionaly, provide additional information about the influence that enteroparasite could have in a malaria vivax development essential for diagnostic and preventive strategies.

## Conclusion

The malaria by *P*. *vivax*is endemic in the region studied and its coinfection with intestinal parasitosis is present in the municipality. Fecal findings demonstrate a diverse enteroparasitic prevalence among the groups, highlighting the particularities and geographic aspects related to the determinants of health in the border area. In addition to this context, there was a predominance of male individuals in the adult age group people with low purchasing power and who were engaged in activities related to goldmining. Hemoglobin levels appeared to be strongly related to malaria infection, especially in the context of concomitant infections, with coinfected individuals more likely to be anemic than single infected malaria patients. In this study we did not observe the influence of coinfection on the acquisition of IgG antibodies against PvMSP119, as well as on the profile of the cytokines that characterize the Th1 and Th2 patterns. However, the co-infection between *Plasmodium vivax* and enteroparasitoses increased levels of TNF-α, and IL-10 in comparison to those with single malarial infection. Longitudinal studies including a greater number of patients are needed to better characterize the potential effects of this coinfection in the Brazilian Amazon population’s immune response.

## Supporting information

S1 Table Epidemiological and hematological dataset. (XLSX)

S2 Table Epidemiological and hematological dataset. (DOCX)

S1 Fig Dataset MSP119. (XLSX)

S2 Fig Dataset cytokines. (XLSX)
