# Supplementary material for: Enteroparasite and vivax malaria co-infection on the Brazil-French Guiana border: Epidemiological, haematological and immunological aspects
Source: PLoS One. 2018 Jan 2;13(1):e0189958. doi: 10.1371/journal.pone.0189958 (PMC5749708; doi:10.1371/journal.pone.0189958)
Supplement: S2 Table — (DOCX) [file pone.0189958.s002.docx]

**Tables made through (S1 Table. Epidemiological and hematological dataset.)**

**Table 1. Distribution and Number of Individuals among Groups and Subgroups According to the Malaria and Intestinal Parasite Diagnosis.**

| **Groups** | **Subgroups** | **Description** | **n** | **%** |
| --- | --- | --- | --- | --- |
| Malaria (M) |  | Individuals infected with *Plasmodium vivax* only | 30 | 6.9 |
| Co-infected (CI) | Helminths (H) | Individuals co-infected with *Plasmodium* and helminths only (H) | 54 | 12.2 |
|  | Protozoa (P) | Individuals co-infected with  *Plasmodium*  and protozoa (P) only | 39 | 8.9 |
|  | Helminths + Protozoa (P+H) | Individuals co-infected with *Plasmodium* and helminths + protozoa (P+H) | 24 | 5.4 |
| Total (CI) |  |  | 117 | 26.5 |
| Enteroparasite (E) | Helminths (H) | Individuals infected with helminths only (H) | 63 | 14.2 |
|  | Protozoa (P) | Individuals infected with protozoa only (P) | 68 | 15.4 |
|  | Helminths + Protozoa (P+H) | Individuals infected with helminths and protozoa (P+H) only | 12 | 2.8 |
| Total (E) |  |  | 143 | 32.4 |
| Endemic Control (EC) |  | Individuals negative for malaria and intestinal parasite diagnosis | 151 | 34.2 |
| Total |  |  | 441 | 100 |

Groups: malaria (M), co-infected (CI), enteroparasite (E) and endemic control (EC).

Subgroups CI and E: helminths (H), protozoa (P) and association of helminths and protozoa (P+H).

**Table 2. Epidemiological and Haematological Data for the Studied Groups.**

|  | Malaria-Positive  N=147 | | | |  | Malaria-Negative  N=294 | | | | |
| --- | --- | --- | --- | --- | --- | --- | --- | --- | --- | --- |
|  | Malaria (M)^a^  N=30 | | Co-infected (CI)^b^  N=117 | |  | Enteroparasites (E)^c^  N=143 | | | Endemic control (EC)^d^  N=151 | |
| Category n (%) | |  | |  | | |  |  | |  |
| Male | | 23 (77) | | 70 (60) | | |  | 56 (39) | | 88 (58) |
| Female | | 7 (23) | | 47 (40) | | |  | 87 (61) | | 63 (42) |
| Age | | 29 (12–55) | | 29 (7–66)^d*^ | | |  | 25 (8–74)^d*^ | | 19 (10–60) |
| RT | | 29 (12–79)^d*^ | | 26 (7–66)^d*^ | | |  | 25 (8–65)^d*^ | | 19 (10–60) |
| NPE | | 4 (2–10) | | 5 (0–17) | | |  | 4 (0–17) | | 4 (1–16) |
| PLM | | 9 (5–14) | | 8 (0–15)^c*d*^ | | |  | 9 (0–17) | | 9 (4–18) |
| Haemoglobin (g/dL) | | 13.8 (11.7–17)^b*c*^ | | 13.2 (7.7–18.2)^d*^ | | |  | 12.4 (9.4–16.7)^d*^ | | 13.7 (9.4–16.7) |
| Anaemia (%) | | 10% (3/30) | | 30.8% (36/117)^d*^ | | |  | 43.3% (62/143) | | 10.6% (16/151) |
| Parasitaemia (par./𝜇L) | | 2750 (60–16.000)^b*^ | | 1000 (25–30.000) | | |  | (--) | | (--) |
| Gametocytes | | 250 (0–6000) | | 70 (0–6000) | | |  | (--) | | (--) |

n (%): number of samples (percentage) in each category

Values expressed as medians (25–75%): age, residence time (years) in Oiapoque (RT), number of previous malaria episodes (NPE), period (months) since last malaria (PLM), haemoglobin levels (g/dL), parasitaemia (parasites/L) and gametocytes.

The differences between the groups with regard to their age, RT, NPE, PLM and haemoglobin were calculated using Tukey's test and based on a one-way ANOVA.

Individuals with haemoglobin levels ≤ 13 g/dL for men, ≤ 12 g/dL for women and children ≥ 11 g/dLwere considered to have anaemia, according to the Kruskal-Wallis-Dunn test.

Differences in parasitaemia and gametocytes between the malaria and co-infected groups were calculated using the Wilcoxon Mann-Whitney test.

^a^Difference between indicated group and the malaria group

^b^Difference between the indicated group and the co-infected group

^c^Differences between the indicated group and the enteroparasite group

^d^Differences between the indicated group and the endemic control

Statistical differences in epidemiological parameters were expressed as * p < 0.05.

**Statistical analysis performed through (S1 Table. Epidemiological and hematological dataset.)**

**Analysis of Variancia_Tukey_Age**

H0: Age does not influence the difference between the sample means of the studied groups of Malaria, Coinfected, Enteroparasites and control group. μ1 = μ2 = μ3

H1: Age influences the difference between the sample means in the studied groups of Malaria, Coinfected, Enteroparasites and control group, with a difference between at least two means μ1 ≠ μ2

FONTES DE VARIAÇÃO GL SQ QM

Tratamentos 3 24.9 e+02 831.606

Erro 437 70.7 e+03 161.705

F = 5.1427

(p) = 0.0021

Média (Coluna 1) = 24.3179

Média (Coluna 2) = 30.4000

Média (Coluna 3) = 29.8291

Média (Coluna 4) = 28.2448

Tukey: Diferença Q (p)

Médias ( 1 a 2) = 6.0821 3.3839 ns

Médias ( 1 a 3) = 5.5112 4.9764 < 0.01

Médias ( 1 a 4) = 3.9269 3.7427 < 0.05

Médias ( 2 a 3) = 0.5709 0.3103 ns

Médias ( 2 a 4) = 2.1552 1.1936 ns

Médias ( 3 a 4) = 1.5843 1.4134 ns

From the Tukey test, there were differences between the sample mean values for Age between the Coinfected and Enteroparasite groups (Group 3 and 4, <0.01 and <0.05), respectively, when compared with the control group, but there was no difference between Malaria (group 1). There was also no difference between the malarial and coinfected groups (groups 2 and 3), malaria and parasites (groups 2 and 4) and coinfected and parasites (groups 3 and 4).

**Analysis of Variancia_Tukey_Time of residence**

H0: The residence time does not influence the difference between the sample means in the studied groups of Malaria, Coinfected, Enteroparasites and control group. μ1 = μ2 = μ3

H1: The residence time influences the difference between the sample means in the studied groups of Malaria, Coinfected, Enteroparasites and control group, with a difference between at least two means μ1 ≠ μ2

FONTES DE VARIAÇÃO GL SQ QM

Tratamentos 3 22.1 e+02 737.058

Erro 437 64.6 e+03 147.725

F = 4.9894

(p) = 0.0025

Média (Coluna 1) = 23.9536

Média (Coluna 2) = 27.6573

Média (Coluna 3) = 28.3590

Média (Coluna 4) = 31.2667

Tukey: Diferença Q (p)

Médias ( 1 a 2) = 3.7037 3.6932 < 0.05

Médias ( 1 a 3) = 4.4053 4.1618 < 0.05

Médias ( 1 a 4) = 7.3130 4.2569 < 0.05

Médias ( 2 a 3) = 0.7016 0.6549 ns

Médias ( 2 a 4) = 3.6093 2.0913 ns

Médias ( 3 a 4) = 2.9077 1.6532 ns

The Tukey's test showed differences between the sample mean values for the residence time between Enteroparasites, Coinfected and Malarious (p <0.05) groups when compared with the control group. However, there were no significant differences between groups: Enteroparasites and coinfected , Enteroparasites and Malaric and Coinfected and Malaric.

**Analysis of Variancia_Tukey_Number of episodes of Malaria**

H0: The malaria episode number does not influence the difference between the sample means of the studied groups of Malaria, Coinfected, Enteroparasites and control group. μ1 = μ2 = μ3

H1: The residence time influences the difference between the sample means in the studied groups of Malaria, Coinfected, Enteroparasites and control group, with a difference between at least two means μ1 ≠ μ2

FONTES DE VARIAÇÃO GL SQ QM

Tratamentos 3 67.752 22.584

Erro 437 42.2 e+02 9.657

F = 2.3386

(p) = 0.0716

By the Tukey's test there were no differences between the sample means for the number of episodes of malaria among the groups studied (p = 0.0716).

**Analysis of Variancia_Tukey_Period of Ultima Malaria**

H0: The period of the last malaria does not influence the difference between the sample means of the studied groups of Malaria, Coinfected, Enteroparasites and control group. μ1 = μ2 = μ3

H1: The period of the last malaria influences the difference between the sample means in the studied groups of Malaria, Coinfected, Enteroparasites and control group, with a difference between at least two means μ1 ≠ μ2

FONTES DE VARIAÇÃO GL SQ QM

Tratamentos 3 142.399 47.466

Erro 437 27.3 e+02 6.236

F = 7.6114

(p) = 0.0002

Média (Coluna 1) = 9.2119

Média (Coluna 2) = 8.8462

Média (Coluna 3) = 7.7949

Média (Coluna 4) = 8.3000

Tukey: Diferença Q (p)

Médias ( 1 a 2) = 0.3658 1.7752 ns

Médias ( 1 a 3) = 1.4170 6.5156 < 0.01

Médias ( 1 a 4) = 0.9119 2.5836 ns

Médias ( 2 a 3) = 1.0513 4.7758 < 0.01

Médias ( 2 a 4) = 0.5462 1.5402 ns

Médias ( 3 a 4) = 0.5051 1.3978 ns

Tukey's test showed differences between the control and coinfected groups (p <0.01) and between Enteroparasites and coinfected groups (p <0.01), but there was no significant difference between the groups. control and enteroparasites, control and malaria, enteroparasites and malaria and coinfected and malarious.

**Analysis of variance_Tukey_Hemoglobin**

H0: Hemoglobin levels did not influence the difference between the sample means of the studied groups of Malaria, Coinfected, Enteroparasites and control group. μ1 = μ2 = μ3

H1: Hemoglobin levels influence the difference between the sample means in the studied groups of Malaria, Coinfected, Enteroparasites and control group, with a difference between at least two means μ1 ≠ μ2

FONTES DE VARIAÇÃO GL SQ QM

Tratamentos 3 106.756 35.585

Erro 437 841.838 1.926

F = 18.4724

(p) = < 0.0001

Média (Coluna 1) = 13.7861

Média (Coluna 2) = 12.7210

Média (Coluna 3) = 12.9171

Média (Coluna 4) = 13.8533

Tukey: Diferença Q (p)

Médias ( 1 a 2) = 1.0651 9.3008 < 0.01

Médias ( 1 a 3) = 0.8690 7.1891 < 0.01

Médias ( 1 a 4) = 0.0672 0.3428 ns

Médias ( 2 a 3) = 0.1961 1.6030 ns

Médias ( 2 a 4) = 1.1324 5.7455 < 0.01

Médias ( 3 a 4) = 0.9362 4.6615 < 0.01

The Tukey test showed differences between the control and enteroparasite (p <0.01), control and coinfected (p <0.01), enteroparasites and malaric (p <0.01) and coinfected and malarious <0.01). However, there were no significant differences between groups: control and malarious and enteroparasites and coinfected.

**HYPOTHESIS TEST: Test t - Two Independent Samples**

**PARASITEMIA**

H0: There is no difference between the parasitemia found in the malarial and coinfected groups: μ1 = μ2;

H1: there is a difference between the parasitemia found in the malarial and coinfected groups: μ1 ≠ μ2;

Decision level: alpha = 0.05.

____________________________________

There is no difference between the parasitemia found in the malarial and coinfected groups: μ1 = μ2 (p = 0.0190).

Resultado Amostra 1 Amostra 2

Tamanho da amostra 30 117

Soma dos Postos (Ri) 2725.0 8153.0

Mediana = 2750.00 1000.00

U = 1250.00

Z(U) = 2.4272

p-valor (unilateral) = 0.0076

p-valor (bilateral) = 0.0152

The U test is statistically significant (p = 0.0152), thus rejecting the null hypothesis and accepting the alternative, with a difference between the parasitemia found in the malarial and coinfected groups.

**The parasitemia differences between the co-infected and malaria groups were calculated using Wilcoxon-Mann-Whitney, a non-parametric test consisting of the evaluation of the difference between two independent samples.**

**GAMETOCYTES**

H0: There is no difference between the quantitative gametocytes found in the malarial and coinfected groups: μ1 = μ2;

H1: there is difference between the quantitative gametocytes found in the malarial and coinfected groups: μ1 ≠ μ2;

Decision level: alpha = 0.05.

____________________________________

There is no difference between the quantitative gametocytes found in the malarial and coinfected groups: μ1 = μ2 (p = 0.2510).

**Resultado Amostra 1 Amostra 2**

**Tamanho da amostra 30 117**

**Soma dos Postos (Ri) 2582.0 8296.0**

**Mediana = 250.00 70.00**

**U = 1393.00**

**Z(U) = 1.7399**

**p-valor (unilateral) = 0.0409**

**p-valor (bilateral) = 0.0819**

The U test was not statistically significant (p = 0.0819), no, there being a difference between the amount of gametocytes found in the malarial and coinfected groups.

**The parasitemia differences between the co-infected and malaria groups were calculated using Wilcoxon-Mann-Whitney, a non-parametric test consisting of the evaluation of the difference between two independent samples.**

ANEMIA

Resultados

H = 9.5849

Graus de liberdade = 3

(p) Kruskal-Wallis = 0.0224

R 1 = 242.5000

R 2 = 1748.0000

R 3 = 3669.0000

R 4 = 1243.5000

R 1 (posto médio) = 80.8333

R 2 (posto médio) = 48.5556

R 3 (posto médio) = 59.1774

R 4 (posto médio) = 77.7188

Comparações (método de Dunn) Dif. Postos z calculado z crítico p

Postos médios 1 e 2 32.2778 1.5836 2.635 ns

Postos médios 1 e 3 21.6559 1.0800 2.635 ns

Postos médios 1 e 4 3.1146 0.1459 2.635 ns

Postos médios 2 e 3 10.6219 1.4945 2.635 ns

Postos médios 2 e 4 29.1632 2.8616 2.635 < 0.05

Postos médios 3 e 4 18.5413 1.9494 2.635 ns

P- Kruskal-Wallis = 0.0224
